# Supplementary material for: Stacking Model‐Based Classifiers for Dealing With Multiple Sets of Noisy Labels
Source: Biom J. 2025 Mar 12;67(2):e70042. doi: 10.1002/bimj.70042 (PMC11898607; doi:10.1002/bimj.70042)
Supplement: Supplementary file 1 — Supporting Information [file BIMJ-67-e70042-s001.zip › density-based-ensemble-model-main/competitors_real_data/netlab-master/netlab-master/help/demgtm2.htm]

Netlab Reference Manual demgtm2


# demgtm2

## Purpose

Demonstrate GTM for visualisation.

## Synopsis

```
demgtm2
```

## Description

This script demonstrates the use of a
GTM with a two-dimensional latent space to visualise data in a higher
dimensional space.
This is done through the use of the mean responsibility and magnification
factors.

## See Also

`demgtm1`, `gtm`, `gtmem`, `gtmpost`

---

**Pages:**
Index


---

Copyright (c) Ian T Nabney (1996-9)
